# Qualitative Job Insecurity and Informal Learning: A Longitudinal Test of Occupational Self-Efficacy and Psychological Contract Breach as Mediators

**DOI:** 10.3390/ijerph16101847

**Published:** 2019-05-24

**Authors:** Anahí Van Hootegem, Hans De Witte

**Affiliations:** 1 Research group for Work, Organisational, and Personnel Psychology, KU Leuven, 3000 Leuven, Belgium; hans.dewitte@kuleuven.be; 2 Optentia Research Focus Area, North-West University, Vanderbijlpark 1900, South Africa

**Keywords:** job insecurity, job features, informal learning, conservation of resources theory, psychological contract theory, occupational health

## Abstract

Current work life has become increasingly turbulent, which has sparked employees’ concern about the loss of valued job features, coined as qualitative job insecurity. No prior research has investigated the relationship between this type of job insecurity and informal learning. However, informal learning might be particularly relevant for qualitatively job-insecure employees, as it might aid them to deal with the incessant changes in their work environment. This study examined whether qualitative job insecurity is associated with lower levels of three types of informal learning activities: information-seeking, feedback-seeking, and help-seeking behavior, and whether these relationships are mediated by a decline in occupational self-efficacy and an increase in psychological contract breach. We employed a three-wave panel design to survey 1433 Belgian employees. Results, by means of cross-lagged structural equation modelling, demonstrated that occupational self-efficacy mediates the relationship between qualitative job insecurity and information-seeking, feedback-seeking from colleagues, and feedback-seeking from one’s supervisor, while psychological contract breach only mediated the relationship between qualitative job insecurity and feedback-seeking from one’s supervisor. Both mediators were not significantly related to help-seeking behavior. This study demonstrates that qualitatively job-insecure employees are less likely to engage in informal learning via a decrease in occupational self-efficacy and an increase in psychological contract breach, thereby becoming even more vulnerable in an increasingly volatile work environment.

## 1. Introduction

Over the past two decades, the labour market has become more volatile, stemming from economical, technological, and societal changes, such as an expansion in global competition, technological developments, and an increase in flexible employment relationships. On the organisational level, these changes have translated themselves in the form of many restructuring initiatives, thereby impacting workers’ employment and working conditions [1]. Consequently, this has left an increasing number of employees worried about the continuity of their work-related future, which is coined as job insecurity [2]. This has sparked a growing research stream, which has mostly directed its attention towards quantitative job insecurity, referring to the perceived threat of losing one’s job as a whole [3]. Conversely, much less research has focused on the qualitative counterpart of job insecurity, which has been defined as the perceived threat of losing valued job features [3]. This type of job insecurity is increasingly relevant in the light of continuous organisational changes, as these might lead to employee concerns about the devaluation of their job quality, without employment being at stake [4].

A number of studies have investigated the consequences of qualitative job insecurity for employee behavior, and have indicated that qualitative job insecurity is related to a decline in organizational citizenship behavior [5] and job performance [6], and an increase in counterproductive work behavior [7]. To the best of our knowledge, however, no research thus far has addressed its relationship with employees’ informal learning behavior. Informal learning might be particularly relevant for qualitatively job-insecure employees, as it might aid them to deal with the incessant changes in their work environment [8]. In line with this, previous research has demonstrated that the acquisition of new knowledge, skills, and abilities functions as a buffer against the stressful consequences of a changing job situation [9]. Prior research findings, however, indicate that employees generally respond to qualitative job insecurity by withdrawing from the job and the organisation [4]. Contradictorily, this might imply that qualitatively job-insecure workers will be less likely to engage in informal learning, even though they might benefit more, thereby becoming more vulnerable in an increasingly turbulent work environment. Therefore, the current study addresses the relationship between qualitative job insecurity and informal learning.

In addition, it is important to investigate the processes that might underlie this relationship. To this end, we build on conservation of resources (COR) theory and psychological contract theory as theoretical lenses that might explain the association between qualitative job insecurity and informal learning. We focus on both theories for two reasons. First, these theories have been shown to be two of the most important frameworks within the job insecurity–outcome relationship [10]. Second, the underlying processes within these frameworks have different emphases. COR theory argues that individuals under stress might not have the resources to deal with stressors, and are, consequently, more vulnerable to losing resources and withdrawing from one’s job [11]. Hence, COR mainly builds on individuals’ stress response, whereas psychological contract theory, in contrast, also pertains to the notion of reciprocity. This social-exchange perspective is relevant within the light of informal learning, as these behaviors are viewed as strategies to successfully perform one’s job within the organisation [12,13]. Therefore, an employees’ exchange relationship with their organisation might also influence the extent to which they engage in behaviors that are instrumental for increased job performance. Consequently, a perceived lack of reciprocity might lead to a decline in informal learning. As each framework provides unique information about the processes that govern the relationship between qualitative job insecurity and informal learning, we simultaneously include two mediating mechanisms, which are each grounded within one of the two frameworks. This allows one to investigate the relative importance of each mediator. We advance occupational self-efficacy (OSE) as a mediating mechanism grounded in conservation of resources theory as it has been put forward as an one of the most important personal resources within this theoretical perspective [14,15], and since prior research findings have demonstrated that this resource is especially important for engagement in learning behavior [16]. We advance psychological contract breach as an operationalisation of psychological contract theory as potential discrepancies in employees’ psychological contract can lead to breach of the contract, and as psychological contract breach (PCB) is one of the most commonly used representations of this framework [17]. 

Our study contributes to the literature in three ways. First, we assess the relationship between qualitative job insecurity, and an outcome to which it has not been linked yet, namely informal learning. By investigating this relationship, we provide valuable information about whether qualitative job insecurity as a stressor also impairs informal workplace learning. Second, research on qualitative job insecurity has so far been mostly cross-sectional (see [4,18] for two exceptions). To address this gap, the current study employs a three-wave longitudinal design, which allows one to investigate the long-term consequences of qualitative job insecurity as well as the directionality of the effects. Third, we examine whether occupational self-efficacy and psychological contract breach might explain the relationship between qualitative job insecurity and informal learning. In doing so, we gain insight into the processes through which qualitative job insecurity might affect employees’ informal learning behavior. Additionally, this allows us to contrast two possible mediators (i.e., occupational self-efficacy and psychological contract breach) and to compare which mechanism has more strength in explaining the qualitative job insecurity–informal learning relationship.

### 1.1. Qualitative Job Insecurity

The conceptualisation of job insecurity as a multidimensional construct was introduced by Greenhalgh and Rosenblatt [2] (p. 441), who stated that “loss of valued job features is an important but often overlooked aspect of job insecurity”. This type of job insecurity was later named by Hellgren and colleagues [3] as qualitative job insecurity, contrasting it with the loss of the job itself (i.e., quantitative job insecurity). Qualitative job insecurity pertains to perceived threats of subjectively important aspects of the job, such as deterioration of salary development, career progress, resources, and working conditions [2]. This type of job insecurity is considered to be an equally substantial stressor as quantitative job insecurity, with adverse consequences for the individual as well as the organisation [19,20]. 

Indeed, previous research has demonstrated that qualitative job insecurity is associated with a decline in employee well-being [19,21], organizational commitment [4], career satisfaction [22], and job performance [5,6,23], and an increase in turnover intentions [3] and counterproductive work behavior [7]. Since no prior studies have examined the relationship with informal learning, we aim to examine the negative implications of qualitative job insecurity for employees’ informal workplace learning.

### 1.2. Informal Learning

Informal learning concerns all learning that is unstructured, occurs through everyday practices, in non-educational settings, and without systematic support to foster learning [24]. Prior research has indicated that employees ascribe up to 90% of their personal development to informal learning, suggesting that the majority of learning in organisations occurs more informally [24,25]. From the organization’s perspective, informal workplace learning fosters the continuous development of workers’ knowledge and skills, thereby contributing to a sustainable competitive position [12]. For employees, engaging in informal learning is an important means to improve their employability and to adjust to new demands in their jobs [13,26]. 

The present study taps into three different forms of informal learning, namely, feedback-seeking, help-seeking, and information-seeking behavior. These are viewed as learning behavior, as prior research has demonstrated that consulting each other, receiving feedback and support, and considering resources such as books and the internet are related to improvements in a range of job-specific and generic competencies [13,27]. Since these forms of behavior occur through everyday practices, in non-educational settings, and without systematic support to promote learning, they can be considered as informal forms of learning [24].

We focus on these types of informal learning for two reasons. First, we expect qualitative job insecurity to have a stronger impact on active learning behavior, as this type of behavior is more impressionable by employees themselves, in contrast to learning that is initiated by others. Active learning behavior refers to all learning that is self-directed and self-initiated by nature [28]. By means of this definition, information, feedback, and help-seeking behavior can be considered as active learning. Second, these types of informal learning have been identified by the literature as important forms of informal learning, as indicated by their representation in informal learning measures (e.g., [12,29]), and their relationships with valuable outcomes such as perceived career development and employability [26,30].

Feedback-seeking behavior refers to the proactive search by individuals for underlying criteria about what is important at work and information about how well they are meeting various work goals [31,32]. Asking for feedback is regarded as an interpersonal learning activity, since it provides workers with information on how to perform effectively, which, in turn, helps individuals to improve learning processes and work achievements [30]. Information-seeking is viewed as an individuals’ proactive search for non-evaluative information that is acquired via non-interpersonal sources such as books and the internet [12,33]. Information-seeking behavior is viewed as informal learning, as it helps employees to deal with a specific problem or question, thereby addressing gaps in their knowledge and improving their performance [34]. Help-seeking behavior, in contrast, is in social interaction with others, and can be defined as the search for others’ assistance, information, advice, or support [35,36]. Help-seeking behavior is considered as an informal learning activity, since help-seeking aids to solve problems, which, consequently, further develops employees’ expertise and job knowledge [37].

Although these constructs all emphasize the proactive search to gain specific resources [30], previous research has demonstrated that they vary in the way in which they are related to antecedents and outcomes (e.g., [8,26,37]) As a consequence, this study separately addresses these concepts rather than employing one holistic measurement.

### 1.3. The Mediating Role of Occupational Self-Efficacy

Using the conservation of resources theory [11] as a guiding framework, we argue that qualitative job insecurity is related to informal learning through a decrease in occupational self-efficacy. COR theory states that psychological stress will occur when individuals experience (1) a loss of resources, (2) a threat to losing resources, or (3) an absence of resource gain following resource investment [38] (p. 341). Job features such as developmental opportunities, social support from colleagues, time for work, and income are considered as resources, and, consequently, qualitative job insecurity can be regarded as the threat of losing valued resources, and therefore stressful [38]. 

COR theory argues that when individuals are experiencing stress, they are more likely to lose other resources, such as occupational self-efficacy [11]. Occupational self-efficacy (OSE) is defined as ‘’the competence that a person feels concerning the ability to successfully fulfil the tasks involved in his or her job [39]’’, and is considered to be a personal resource [38]. Employees’ perceptions of occupational self-efficacy involve the sense of their ability to successfully control and impact their environment, which is at the core of the personal resource construct [40]. Hence, we expect that the threat of degenerating job conditions (i.e., qualitative job insecurity) increases workers’ vulnerability to further resource losses, which will manifest itself in a decline in occupational self-efficacy. In addition, threat of resource loss elicits a defensive posture, which consumes additional resources and hinders the advancement of new resources [41]. As self-efficacy is not a static construct but rather a dynamic set of self-beliefs that is fostered by investment in terms of effort and time [42], qualitative job insecurity might undermine employees’ self-efficacy.

Indirect empirical evidence for the negative impact of qualitative job insecurity on self-efficacy is provided by Kinnunen et al. [43], who found that job insecurity leads to lower levels of self-esteem, a concept that is closely related to, but distinct from, self-efficacy [44,45]. In addition, a recent study found that job insecurity has a negative impact on general self-efficacy [46]. Accordingly, we hypothesize the following:

**Hypothesis 1** **(H1).** Qualitative job insecurity will be negatively related to subsequent occupational self-efficacy, in that higher levels of qualitative job insecurity will be associated with lower levels of occupational self-efficacy.

Social cognitive theory [47] argues that beliefs of personal efficacy represent the core of human agency. If individuals do not believe they have the control to produce results, they will not expend high effort or persist in the face of setbacks [47]. People with high self-efficacy set more difficult goals, put in more effort to reach these goals, and persist in stressful situations or aversive experiences [47,48]. Consequently, highly self-efficacious people have a higher likelihood of engaging in activities and behaviors that foster the accomplishment of their goals, such as engaging in informal learning behaviors. 

This fits the notion of COR theory, which states that individuals with greater resources are more capable of orchestrating resource gain, or conversely, those with fewer resources are less able of resources acquisition [38]. Applied to reduced occupational self-efficacy (OSE) following qualitative job insecurity, this entails that employees with low OSE will be less capable of acquiring additional resources. In addition, the reduction of resources elicits a defensive posture, which consumes additional resources and hinders the advancement of new resources [41]. This entails that individuals low in OSE will scale back from activities that further demand their resources, such as informal learning, as informal learning requires an investment in terms of both energy and time [24]. In line with this, scholars have argued that self-directed behavior requires anticipation and action directed towards future impact, and is thereby likely to deplete resources [49,50].

Prior research findings suggest that self-efficacy is an important precursor to participation in informal learning [51,52], in that employees with low self-efficacy are less likely to engage in informal workplace learning. Therefore, we propose the following hypotheses:

**Hypothesis 2** **(H2).** Occupational self-efficacy is positively related to subsequent information-seeking (H2a), feedback-seeking (H2b), and help-seeking (H2c) behavior, in that lower levels of occupational self-efficacy will be associated with lower levels of all three types of informal learning.

**Hypothesis 3** **(H3).** Occupational self-efficacy will mediate the relationships between qualitative job insecurity and information-seeking (H3a), feedback-seeking (H3b), and help-seeking (H3c) behavior, in that negative indirect effects will exist between qualitative job insecurity and all three types of informal learning via occupational self-efficacy.

### 1.4. The Mediating Role of Psychological Contract Breach

A large body of job insecurity research has been grounded in social exchange theories, such as psychological contract theory [10]. Within this framework, we build on PCB as an explaining mechanism for the relationship between qualitative job insecurity and informal learning behavior. The psychological contract refers to “the idiosyncratic set of reciprocal expectations held by employees concerning their obligations and their entitlements” [53] (p. 698). When one or both parties feel that the other party did not fulfil his/her promises, psychological contract breach occurs [54]. Consequently, qualitative job insecurity might pertain to unfulfilled promises regarding job features such as career progress, status, and autonomy [2]. In a study in which human resource managers from different organisations were interviewed, Rousseau [55] demonstrated that promises about promotion opportunities, training, career development, pay, long-term job security, and social support were the most commonly mentioned employer obligations. In line with this, Kickul [56] found that broken promises regarding pay package, opportunities for personal growth, increasing responsibility, and opportunities for promotion were the most frequently rated forms of psychological contract breach. Since prior research suggests that valued job features are part of the psychological contract, employees might view qualitative job insecurity as a violation of the employer’s obligations, thereby leading to perceived breach of the psychological contract [57]. In line with this, prior research has demonstrated a positive relationship between qualitative job insecurity and PCB [23]. Consequently, our hypothesis is as follows:

**Hypothesis 4** **(H4).** Qualitative job insecurity will be positively related to subsequent psychological contract breach, in that higher levels of qualitative job insecurity will be associated with higher levels of psychological contract breach.

Breach of the psychological contract may be viewed as a stressor, as a meta-analysis on the consequences of PCB concluded that affective reactions played a central role in explaining the impact of PCB on work-related outcomes [58]. In line with this, previous research has demonstrated that psychological contract breach leads to work-related and general strain reactions, in which employees withdraw from work and distance themselves from the stressor [57,59]. As informal learning is strongly interwoven with one’s job, employees might also disengage from this aspect of their work.

Another aspect of this stress response is that employees may attempt to restore the imbalance in the employment relationship by calculating their investments and decreasing their efforts [57]. As a result, employees might be less inclined to take steps to improve their functioning. A number of studies have demonstrated a positive relationship between engaging in feedback-seeking, information-seeking, and help-seeking behavior and higher performance [60,61,62]. As engaging in informal learning is a means of personal development as to better fit the organisation and acquire the skills necessary to do one’s job effectively [29], we argue that a decrease in effort might manifest itself in a decline in informal learning. 

Indeed, Pierce and Maurer [63] showed that employees may engage in learning behavior to benefit the organisation, to the extent that a positive exchange relationship exists. As PCB entails a negative exchange relationship, this might contribute to a decrease in informal learning. Prior research findings provide further support for this premise, suggesting that a positive attitude towards the organisation encourages employees to engage in informal learning and increase their knowledge and skills [64,65]. In keeping with the aforementioned theoretical and empirical arguments, we hypothesize the following:

**Hypothesis 5** **(H5).** Psychological contract breach is negatively related to subsequent information-seeking (H5a), feedback-seeking (H5b), and help-seeking (H5c) behavior, in that higher levels of psychological contract breach will be associated with lower levels of all three types of informal learning.

**Hypothesis 6** **(H6).** Psychological contract breach will mediate the relationships between qualitative job insecurity and information-seeking (H6a), feedback-seeking (H6b), and help-seeking (H6c) behavior, in that negative indirect effects will exist between qualitative job insecurity and all three types of informal learning via psychological contract breach.

### 1.5. Conceptual Model 

**Figure 1:**
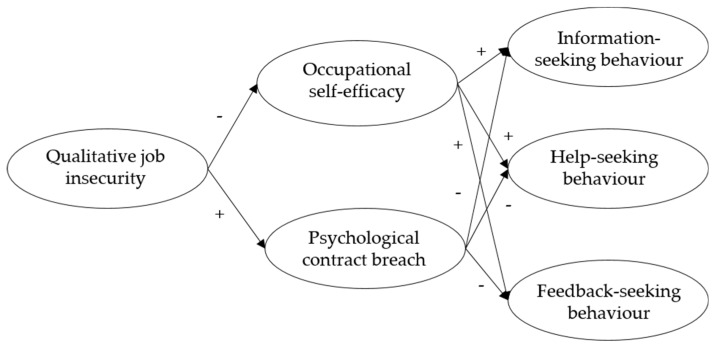
Theoretical model.

Taken together, we hypothesize a parallel multiple mediator model in which occupational self-efficacy and psychological contract breach function as the mediating variables. We expect that both mediations result in a negative indirect effect. By simultaneously including both mediators, we provide information about the relative importance of each pathway. This allows one to establish which processes have more strength in explaining the relationship between qualitative job insecurity and informal learning. Figure 1 provides an overview of the theoretical model and its hypotheses.

## 2. Materials and Methods 

### 2.1. Participants and Procedure

Wave 1 of the survey was organised in June 2017 in one Belgian hospital. This organizational context is specifically relevant to study qualitative job insecurity, as prior research has indicated that many health care institutions are confronted with austerity measures, often forcing them to engage in restructuring [66]. In the hospital where the data of the current study were collected, there have also been austerity measures in the past five years. These measures have led to a cut in hospital staff among the support department. Although care and nursing staff have quantitatively secure job positions, these cuts have led to alterations in their jobs, such as an increase in workload or a decrease in the frequency of meetings. These changing job conditions might have sparked qualitative job insecurity. In line with this, Burke and colleagues [66] demonstrated that hospital staff reported a relatively large number of restructuring and downsizing initiatives within the past year, which was related to higher levels of perceived job insecurity. Furthermore, a study among nurses has shown that more than half of the participants were concerned about their qualitative job insecurity [67]. Lastly, a study in a private hospital setting found that qualitative job insecurity significantly contributed to higher levels of anxiety and depression [68]. 

Data were collected via an online questionnaire, for which participants received an invitation to their work e-mail address. The purpose of the study, the anonymous processing of the data, and the voluntary participation were all emphasized in the introduction of the survey. To abate the attrition rate, however, we raffled five multimedia vouchers of €50 among employees who participated in all three waves. In total, 2299 employees were invited to participate in the study, of which 1502 workers filled in the questionnaire at time 1 (T1), yielding a response rate of 65%. All of the respondents who participated in this wave were invited to participate at time 2 (T2) and time 3 (T3), which took place in December 2017 and June 2018, respectively (a time lag of six months). A total of 723 employees filled in the questionnaire at T2 (response of 48%, relative to T1), and 655 participants responded at T3 (response of 44%, relative to T1). We removed all participants who indicated they experienced job transitions during the study (*n* = 69), since these changes may affect the nature of the cross-lagged relationships [69]. This resulted in a final sample of 1433 employees who participated in the survey at least once over the three time points: 42.7% (*n* = 612) only responded at T1, 15.5% (*n* = 222) completed the survey at T1 and T2, 10.7% (*n* = 154) of the participants filled in the questionnaire at T1 and T3, and 31,1% (*n* = 445) participated in every wave. All of these participants were included in the data analyses using maximum likelihood estimations with robust standard errors, thereby reducing the likelihood of biases due to selective attrition [70]. 

Participants were mostly nursing staff (53.3%), followed by administrative staff (14.2%; e.g., clerical staff, bookkeepers), medical-technical staff (9%; e.g., pharmacists, lab technicians), paramedical staff (7.5%; e.g., physical therapists, dieticians), maintenance staff (6.9%; e.g., cleaners, warehouse(wo)men), policy support staff and management (2.9%, e.g., HR recruiters, paralegals), psycho-social staff (2.7%; e.g., psychologists, welfare officers), patient care assistants (2%), and higher management (1.4%). The average sample age was 41.02 years (SD = 10.78), and participants were predominantly female (81%). The vast majority of the sample had a permanent contract (92%). Approximately 4% of the employees did not receive a degree of secondary education, 16% obtained a degree of secondary education, 67% had a bachelor’s degree, and 13% had a master’s degree or a PhD. The respondents’ mean organisational tenure was 14.32 years (SD = 10.98). 

Little’s missing completely at random (MCAR) test was conducted to check for systematic dropout. We used the variable means of the key study variables at all time points (i.e., qualitative job insecurity, occupational self-efficacy, psychological contract breach, feedback-seeking, help-seeking and information-seeking behavior). The results suggested that the data were MCAR, χ^2^(256) = 292.051, *p* > 0.05. Hence, we employ Mplus’ full information maximum likelihood (FIML) estimation, as this has been shown to generate unbiased estimates under MCAR conditions [71].

### 2.2. Measures

#### 2.2.1. Qualitative Job Insecurity

Qualitative job insecurity was measured with four items that measure similar features to the items of De Witte et al. [19]. This measure assesses the extent to which employees feel insecure about the characteristics and conditions of their job. The items were “I am worried about how my job will look like in the future”, “I think my job will change for the worse”, “I feel insecure about the characteristics and conditions of my job in the future”, and “Chances are, my job will change in a negative way”. Participants responded using a five-point Likert-scale ranging from 1 (strongly disagree) to 5 (strongly agree). This scale has been successfully used in previous studies (e.g., [18,72]). The internal consistency reliability of this scale was α = 0.86 for T1, α = 0.89 for T2, and α = 0.88 for T3.

#### 2.2.2. Occupational Self-Efficacy

We measured occupational self-efficacy using three items from the short version of the occupational self-efficacy scale of Rigotti, et al. [39]. Respondents had to indicate their agreement on a five-point Likert-scale. This scale probes into employees’ belief that they can successfully execute their job. A sample item is “I feel prepared for most of the demands in my job”. The internal consistency reliability of the scale ranged from 0.69 < α < 0.74 across the three waves.

#### 2.2.3. Psychological Contract Breach 

Breach of the psychological contract was assessed using a four item scale developed by Robinson and Morrison [54], and was rated on a five-point Likert-scale ranging from 1 (strongly disagree) to 5 (strongly agree). This scale taps into employees’ perception of the extent to which the organisation has failed to fulfil its promises [73]. A sample item is “My employer has broken many of its promises to me even though I’ve upheld my side of the deal”. The Cronbach’s α ranged between 0.92 at T1 and T2, and 0.90 at T3.

#### 2.2.4. Informal Learning

Three types of informal learning were assessed, all of which were measured on a five-point Likert-scale ranging from 1 (strongly disagree) to 5 (strongly agree). 

Information-seeking behavior. Information-seeking behavior was measured by a scale of Holman et al. [74], and consists of three items. This scale measures employees’ proactive search for information to address gaps in their knowledge (e.g., “I try to understand something better by locating and studying a relevant document”). Cronbach’s alpha was 0.86 at T1 and T2, and 0.87 at T3.

Feedback-seeking behavior. This scale was measured using four items of van Woerkom and Croon’s [32] asking for feedback scale. Two items tap into employees’ search for feedback from their supervisor, while the remaining two items refer to asking feedback from colleagues (e.g., I ask my supervisor for feedback; I ask my colleagues for feedback). The internal consistency of the scale was α = 0.78 at T1, α = 0.79 at T2, and α = 0.76 at T3.

Help-seeking behavior. Help-seeking behavior was evaluated via the two-item scale developed by Froehlich, Beausaert, and Segers [33]. The scale includes items about the extent to which employees ask help from others to solve a specific problem (e.g., “If I were having trouble understanding something at work I would ask someone who could help me understand the general ideas”). The Cronbach alpha coefficient for the scale was 0.68 at T1, 0.69 at T2, and 0.62 at T3.

#### 2.2.5. Control Variables

Since previous research findings have demonstrated that gender (e.g., [30]), age (e.g., [26]), and educational level (e.g., [8]) are associated with the different types of informal learning; we accounted for the possibility that any of the observed relationships may be inflated due to these variables. Hence, gender (0 = male; 1 = female), age (in years), and educational level, recoded into two dummy variables with bachelor’s degree as the reference group (i.e., ‘secondary education degree’: 0 = bachelor’s or master’s degree; 1 = no degree or secondary education degree, and ‘master’s degree’: 0 = no degree, secondary education degree, or bachelor’s degree; 1 = master’s degree or PhD), were included as control variables, thereby following the recommendation of Becker [75] to involve covariates that are likely to relate to the dependent variable.

### 2.3. Analysis Strategy

To investigate the quality of the measurement model, we conducted confirmatory factor analysis (CFA) to test its fit to the data. First, we established the fit of the hypothesized six-factor model, in which all qualitative job insecurity items, occupational self-efficacy items, psychological contract breach items, information-seeking items, feedback-seeking items, and help-seeking items loaded on their respective latent factor at every time point. Item residuals were allowed to correlate with those of corresponding items at previous or consecutive waves. This model was then compared with three alternative models, that is, a four-factor model (in which all types of informal learning load on one factor), a seven-factor model (where feedback-seeking from colleagues and feedback-seeking from the supervisor are divided into two factors), and a one-factor model. 

Choosing the best measurement model from this sequence, we assessed whether the scales showed measurement invariance across time, which was necessary to examine whether the meaning of the constructs had changed over the different time points [76]. The initial measurement model (i.e., configural invariance model; no other constraints than a scale-setting constraint) was compared to a sequence of models, in which increasing restrictions are imposed. The unconstrained model was first compared to a metric invariance model (i.e., factor loadings equal across time), which was subsequently compared to a strong invariance model (i.e., factor loadings and intercepts were constrained to be equal across time), which, in turn, was contrasted with a strict invariance model (i.e., factor loadings, intercepts, and residual variances were fixed to be equal across all waves), which was, lastly, compared with a full invariance model (i.e., loadings, intercepts, residual variances, and correlations between item residuals at adjacent time waves are fixed equal over time) [77].

Next, we tested our hypotheses using a cross-lagged panel design. In addition, we assessed whether the direction of the relationships was as hypothesized. Four different structural models were examined: (i) a stability model with autoregressive paths between the same measurements across waves, (ii) a normal causation model with the proposed causal paths, (iii) a reversed causation model, and (iv) a reciprocal causation model including all paths from the normal causation model and the reversed causation model. The covariates were included in all of the aforementioned models. The normal causation model included direct paths from qualitative job insecurity at T1 to all types of informal learning at T3, and vice versa in the reversed causation model (i.e., from informal learning at T1 to qualitative job insecurity at T3). These direct effects were added since an indirect effect should be assessed controlling for the direct relationship between the independent and the dependent variable [78].

In a final step, we assessed whether the best fitting structural model of this sequence was invariant across time. First, we tested whether the fit of the structural model decreased when fixing the autoregressive paths equal across time. Subsequently, we fixed the cross-lagged paths from the predictor to the mediator, after which the cross-lagged paths between the mediator and the outcomes were fixed equal, thereby providing information as to whether the corresponding paths between the constructs are stable over time. Choosing the best model from this comparison, we performed bootstrap analysis (5000 resamples) to provide confidence intervals (CIs) for the indirect effects. The test of our hypotheses is based on this model. 

All of the analyses were computed using Mplus 7.0 [79] (Mplus, Los Angeles, CA, USA). The goodness of fit of the models was evaluated using several fit indices, more specifically, the Comparative Fit Index (CFI) [80], the Tucker-Lewis Index (TLI) [81], the root mean squared error of approximation (RMSEA) [82], and the standardized root mean squared residual (SRMR) [81]. Following Hu and Bentler’s [81] recommendations for fit index cut-off criteria, CFI and TLI of at least 0.95 indicate a good fit, whereas for RMSEA and SRMR, values below 0.06 and 0.08, respectively, point to a good fit. Competing models were compared by means of the Satorra–Bentler scaled difference chi-square test [83]. However, as χ^2^ statistics are sensitive to sample size and to violation of the normality assumption, their significance should not automatically lead to the rejection of a model [76,84]. Alternatively, scholars have proposed to use critical values based on changes in goodness-of-fit indexes, such as the difference in the CFI, for which a cut-of ∆CFI 0.01 has been recommended [84,85]. Therefore, these criteria are also employed for model evaluation.

## 3. Results

Table 1 shows the Pearson’s correlations, means, and standard deviations of the variables of primary interest in this study.

### 3.1. Measurement Model and Measurement Invariance

Table 2 presents an overview of the fit indices of the different model comparisons. The hypothesized measurement model, which consisted of six factors at each wave, showed a relatively good fit to the data, and a significantly better fit than the one-factor model (all items load on one factor at each wave) and the four-factor model (all informal learning items load on one factor at each wave), as indicated by Satorra–Bentler scaled chi-square difference test. This model was then compared to an alternative seven-factor model (feedback seeking divided in feedback seeking from supervisor and from colleagues at each wave). The Satorra–Bentler chi-square difference test indicated that the seven-factor model significantly outperformed the hypothesized measurement model. In addition, this model provided a good fit to the data, as all fit indices adhered to the cut-off criteria suggested by Hu and Bentler [81] (χ^2^ (1440) = 2313.664, CFI = 0.968, TLI = 0.961, RMSEA = 0.021, SRMR = 0.037). All of the items had significant factor loadings, ranging from λ = 0.57 to 0.97. As a consequence, we chose the seven-factor model over the hypothesized six-factor model. 

Subsequently, the measurement invariance of this model was investigated. The Satorra–Bentler scaled chi-square difference test indicated that all of the comparisons in model fit were significant, suggesting non-invariance. However, as we have a large sample (*n* > 200), the ∆χ^2^ is likely to be biased against invariance, entailing that a trivial discrepancy might lead to an unjust rejection of a model [76]. Hence, we used the threshold of ∆CFI < 0.01 as a criterion for measurement invariance. Since constraining the factor loadings, the intercepts, the residual variances, and the correlations between item residuals at adjacent time waves equally across time did not violate the threshold of 0.01, full measurement invariance across time was assumed.

### 3.2. Test of the Hypotheses and Time Invariance

Table 3 provides an overview of the results of a comparison of competing structural models. To test the hypothesized direction of the cross-lagged relationships, we compared a sequence of four structural models. The results showed that the normal causation model had a significantly better fit than the stability model (χ^2^ (1865) = 3529.514, Satorra–Bentler adjusted ∆χ^2^ = 106.515, ∆df = 25, *p* < 0.001). While the reversed causation model provided a better fit compared to the stability model, the reciprocal causation model did not present a significantly better fit than the normal causation model (χ^2^ (1841) = 3504.213, Satorra–Bentler adjusted ∆χ^2^ = 26.607, ∆df = 24, *p* > 0.05). In addition, none of the added reversed causation pathways were significant. As the reciprocal causation model did not significantly improve model fit, we chose the more parsimonious model, that is, the normal causation model, over the reciprocal model. Hence, no evidence was found for reversed effects.

**Figure 2:**
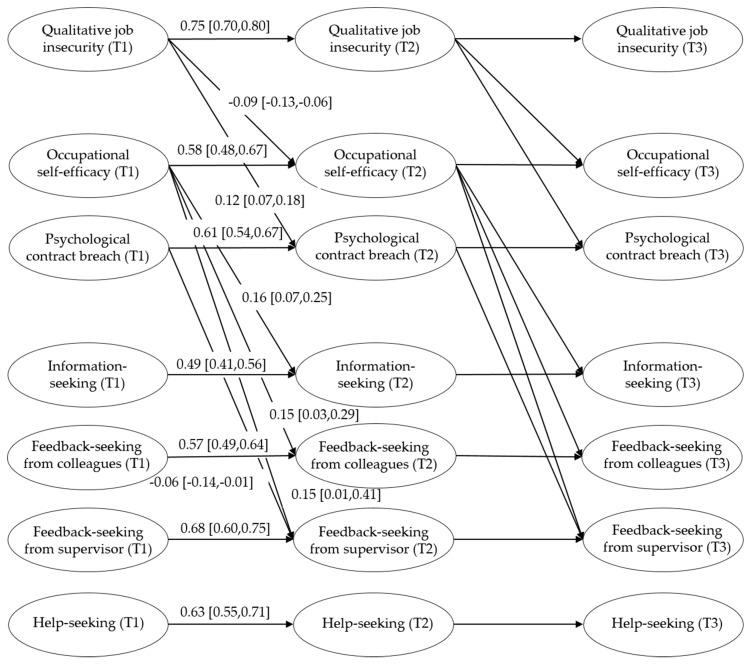
Structural equation model with unstandardized path coefficients and confidence intervals. Control variables and insignificant pathways are omitted for clarity. Coefficients were fixed to be equal across time, and, consequently, coefficients between T2 and T3 are also omitted for clarity.

In a subsequent series of analyses, we examined the time invariance of the normal causation model, as to obtain information about the stability of the pathways over time. The fit of the model did not decrease when we fixed the autoregressive paths equal across time (see Table 3). Constraining the cross-lagged pathways from qualitative job insecurity to both mediators, or from the mediators to the different types of informal learning, also did not compromise model fit (Satorra–Bentler adjusted ∆χ^2^ = 0.121, ∆df = 2, *p* > 0.05; and Satorra–Bentler adjusted ∆χ^2^ = 24.812, ∆df = 8, *p* > 0.05, respectively). This supported the stability of the relationships across time. Our hypothesized effects were examined based on this final model, which are summarized in Figure 2.

Our results showed that qualitative job insecurity is negatively related to occupational self-efficacy, thereby providing support for Hypothesis 1. Furthermore, occupational self-efficacy was, as hypothesized, positively related to information-seeking, feedback-seeking from colleagues, and feedback-seeking from one’s supervisor. Thus, Hypothesis 2a and 2b were supported. However, no significant relationship was found between occupational self-efficacy and help-seeking behavior, and, consequently, Hypothesis 2c could not be supported. This also resulted in the rejection of Hypothesis 3c, which assumed an indirect effect from qualitative job insecurity to help-seeking through occupational-self efficacy. The results did show that there were significant indirect effects of qualitative job insecurity at T1, via occupational self-efficacy at T2 on information-seeking (B = −0.014, bootstrapped 95% CI [−0.028, −0.007]), feedback-seeking from colleagues (B = −0.014, bootstrapped 95% CI [−0.032, −0.002]), and feedback-seeking from one’s supervisor at T3 (B = −0.014, bootstrapped 95% CI [−0.031, −0.003]), supporting Hypothesis 3a and 3b.

With regard to our hypotheses on psychological contract breach, the results demonstrated that qualitative job insecurity is positively associated with psychological contract breach, lending support to Hypothesis 4. In contrast to Hypothesis 5a and 5c, the results did not support a significant relationship between psychological contract breach and information-seeking or help-seeking behavior. This also resulted in rejection of Hypothesis 6a and 6c, which expected that the relationship between qualitative job insecurity and information-seeking and help-seeking behavior would be mediated by psychological contract breach. The result partially confirmed Hypothesis 5b, which proposed a negative relationship between psychological contract breach and feedback-seeking behavior. While we did not find a significant association between psychological contract breach and feedback-seeking from colleagues, we did find a negative effect on feedback-seeking from one’s supervisor. There was also a significant indirect effect of qualitative job insecurity at T1, via psychological contract breach at T2 on feedback-seeking from one’s supervisor at T3 (B = −0.008, bootstrapped 95% CI [−0.02, −0.002]), partially supporting Hypothesis 6b. None of the direct pathways from qualitative job insecurity at T1 to all types of informal learning at T3 were significant.

## 4. Discussion

The aim of the current study was to investigate the relationship between qualitative job insecurity and informal learning, and to advance occupational self-efficacy (OSE) and psychological contract breach (PCB) as explaining mechanisms within this relationship. We considered four different forms of informal learning, namely, information-seeking, feedback-seeking from one’s supervisor, feedback-seeking from colleagues, and help-seeking behavior.

The mediating role of occupational self-efficacy was grounded in conservation of resources (COR) theory, which states that individuals under stress are more vulnerable to a cascading of ongoing resource loss [86]. In line with this, we expected the work stressor qualitative job insecurity to diminish employees’ OSE, as this is considered as a personal resource [38]. Furthermore, COR argues that individuals often resort to the conservation of remaining resources by decreasing their performance efforts [86], which we expected to translate itself in a decrease in informal learning. Our findings partially provided support for these hypotheses, as qualitative job insecurity was related to lower levels of information-seeking, and feedback-seeking from one’s supervisor and colleagues through a decline in occupational self-efficacy.

The path from OSE to help-seeking behavior, however, was found not to be significant. This relationship has been rarely investigated, and the few scholars that have addressed this pathway tended to have opposite predictions regarding the nature of the relationship. On the one hand, researchers have argued that there is a positive link between self-efficacy and help-seeking, in which highly efficacious employees are more likely to seek help because of the lower psychological costs associated with this behavior [87]. Self-efficacious individuals’ feelings of competence might be less easily threatened by asking for help, which entails that help-seeking poses less long-term risks to the self-image of employees with high levels of self-efficacy [88]. Hence, these employees might seek more help because of the lower psychological costs associated with this behavior. On the other hand, it has been suggested that self-efficacy negatively influences help-seeking behavior, as employees with high levels of self-efficacy have a greater belief in their ability to master their work, and, consequently, will persist longer on tasks without asking aid from others [89]. It is possible that the relationship between OSE and help-seeking is more complex in nature than we anticipated, and that these opposite relationships provoke an insignificant effect. Future studies might benefit from further investigating these pathways to disentangle the nature of this relationship.

Concerning the mediating role of psychological contract breach, the hypotheses were underpinned by psychological contract theory, which argues that qualitative job insecurity might be viewed as a breach of the organisation’s promises [90]. Along these lines, we expected qualitative job insecurity to relate to an increase in psychological contract breach. In response to this breach, employees might attempt to restore the imbalance in the employment relationship by decreasing their effort, which we hypothesized to reflect itself in a lower inclination to engage in informal learning. The findings partially supported the hypotheses, as there was a negative indirect effect of qualitative job insecurity on feedback-seeking from supervisors via psychological contract breach.

Contrary to our expectations, we did not find a relationship between PCB and the other forms of informal learning. It is possible that we solely found a significant relationship between PCB and feedback-seeking from one’s supervisor due to the source of the feedback. Supervisors act as important representatives of the organisation by carrying out its promises, and, consequently, breach of the psychological contract might cause workers to distance themselves from their supervisors [91]. In line with this, prior research has demonstrated that employees can derive their psychological contract from multiple sources, including first line supervisors [17,92]. In addition, a study by Lapointe and colleagues [91] demonstrated that psychological contract breach was related to lower levels of affective commitment towards the supervisor. Along these lines, PCB might especially impact informal learning that requires interaction with one’s supervisor and fewer other forms of informal learning behavior.

In the present study, we also aimed to gain information on the relative importance of occupational self-efficacy and psychological contract breach as explaining mechanisms stemming from different theoretical frameworks, that is, conservation of resources theory and psychological contract theory, respectively. Based on our results, conservation of resources theory seems to shed more light on the relationship between qualitative job insecurity and informal learning than psychological contract theory, since OSE accounted for stronger, and more, indirect effects of qualitative job insecurity on informal learning. Thus, it appears the aforementioned relationship is more so grounded in the need to conserve resources than in principles of social-exchange. This entails that qualitatively job-insecure employees rather withdraw from learning because they enter a defensive mode to preserve their resources than because they experience a lack of reciprocity in the exchange relationship [15]. Psychological contract breach, however, also accounted for one significant indirect effect from qualitative job insecurity to feedback-seeking from one’s supervisor, indicating that a perceived imbalance in the employment relationship might be an important explaining mechanism pertaining to informal learning that includes one’s supervisor. In sum, our results indicate that both mechanisms are supplementary and offer a unique lens in understanding the relationship between qualitative job insecurity and informal learning.

### 4.1. Limitations and Further Research

As with any study, the current study also has a few limitations that deserve mentioning. Specifically, the time lags between the measurements were not theoretically based. To the best of our knowledge, however, no research has thus far investigated the desirable length of time when examining reactions to job insecurity. Since the best time lag is highly dependent on the nature of the stressor, future research may want to investigate the preferred time lags for cross-lagged relationships between job insecurity and various outcomes [4,93].

Furthermore, all of our data were based on self-report measures, which might have contributed to common method bias. A recent review on common method bias, however, concludes that the danger that common method variance poses to validity is very limited, suggesting that the issue has received more attention than necessary [94]. Furthermore, we reduced the small risk of common method bias by employing a repeated measures design. Additionally, prior research has indicated that the use of a larger number of variables can mitigate the effects of common method variance (CMV) in data, based on studies with an average of 12 variables [95]. Since the present study contained 25 variables, our analyses included enough measures to make inflation from CMV unlikely. These precautions further minimized the risk of common method bias. While the measurements of job insecurity, occupational self-efficacy, and psychological contract breach all require a subjective perspective, future research might benefit from using other-rated measurements of informal learning, such as supervisor or colleague-ratings.

An additional limitation concerns the relatively small effect size of the indirect effects of qualitative job insecurity on informal learning. All of our concepts were relatively stable over time, which entailed that most of the variance of the variables could be explained by the respective previous measurements of these constructs. Yet, the current study still found significant effects after controlling for baseline levels of our variables, which suggests that the studied relationships are relevant to investigate.

Although the present study used a longitudinal design, no causal inferences can be made regarding our results. Future studies could shed more light on the causal nature of the relationships by employing an experimental design in which perceived qualitative job insecurity is manipulated. This, however, would reduce the ecological validity of the results, as a laboratory experiment is the only way to ethically manipulate the experience of job insecurity [96].

Another limitation pertains to the lack of generalisability of our results to occupational groups and sectors that were not present in our sample. Therefore, cross-validation studies in different sectors are necessary to test whether our results are replicable in other settings than a healthcare context.

Furthermore, we concentrated on qualitative job insecurity, as this type of insecurity is highly relevant to investigate within a hospital setting. However, future research may want to simultaneously include quantitative and qualitative job insecurity as to compare the relative contributions of each type of insecurity on informal learning.

Another interesting avenue for future research may be to explore employees’ perceived reasons for job insecurity, as these might influence the ways in which workers respond to job insecurity. This is in line with research by van Vuuren and colleagues, that has demonstrated that employees who are insecure about their job differ considerably in the causes they attribute to their job insecurity concerns [97], and that employee reactions to job insecurity vary depending on their causal attributions [98].

In addition to the perceived cause of job insecurity, future studies may want to tap into the different dimensions of qualitative job insecurity. Whereas the measurement we used solely assessed the overall concern that one’s job might negatively change, it would be interesting to investigate insecurity about specific aspects of one’s job, as individuals may react differently to the deterioration of various job features.

The current study focused on informal learning due to the prevalence and increasing importance of this type of learning for employee development [24]. Learning behavior, however, also comprises of formal learning, which is defined as all designed learning that happens in a structured context deliberately created for that purpose, and that may lead to formal recognitions, such as diplomas or certificates [99]. Hence, future research might benefit from simultaneously considering both formal and informal learning, as to compare the way in which these outcomes are influenced by qualitative job insecurity and its underlying pathways. In addition, future studies could focus on other types of informal learning behavior. We specifically focused on information-seeking, feedback-seeking, and help-seeking behavior, as these have been identified by the literature as eminent forms of informal learning. Nonetheless, research has shown that learning from oneself, such as reflecting on one’s performance and experimenting with new ways of working, are also important dimensions of informal learning [12]. Future research could also take into account these forms of informal learning to compare the effect of qualitative job insecurity on these different output measures.

Future research might also benefit from looking at the investigated relationships from a team-perspective. As healthcare is becoming more and more complex, the demand for collaborative care has increased [100]. This has resulted in a growing number of health care staff working in a team-based structure. Consequently, it might be interesting to investigate whether, and how, qualitative job insecurity climate, referred to as employee perceptions of the level of qualitative job insecurity in their workgroup, influences informal learning [101]. Since team members function as important internal learning networks within organisations, this view may be specifically relevant in relation to informal learning [102].

A promising line of future research could be to explore the investigated relationships from a person-centred view, in which differences among individuals in how qualitative job insecurity relates to informal learning are investigated [103]. Since the research question of the current study pertained to the association between qualitative job insecurity and informal learning, and the relative importance of the mediators in explaining variance in this relationship, a variable-approach was more appropriate for this study. However, in line with Laursen and Hoff [103], we view both person- and variable-centred techniques as complimentary approaches that, together, lead to a more complete understanding of the processes that underlie employee behavior.

Another potential direction for future research could be the inclusion of cost-benefit calculations, as these are often seen as central within social informal learning constructs such as feedback- and help-seeking behavior [35,104]. It is possible that conveying a negative image, for instance, is perceived as specifically costly in light of concerns about job aspects such as career opportunities, pay, or promotion. Therefore, future scholars could be mindful of the way in which qualitative job insecurity influences perceived costs and benefits of informal learning.

### 4.2. Practical Implications

From a practical standpoint, this study has important implications for individuals as well as organisations. Our results suggest that qualitatively job-insecure employees will engage less in informal learning, which might further increase the vulnerable position of these workers. The withdrawal from informal learning might lead to a decline in competencies, knowledge, and skills, which function as especially important resources in an increasingly volatile organisational environment. In terms of organisations, the results indicate that qualitative job insecurity might undermine the extent to which employees keep their knowledge and skill up-to-date and adjust to new work demands, which is particularly important in times of organisational change if organisations want to sustain their competitive position [105].

Therefore, organisations would be well served to invest in interventions that limit the extent to which their workforce experiences qualitative job insecurity or its negative consequences. To prevent qualitative job insecurity from occurring in the first place, organisations might benefit from implementing HRM practices that are aimed at augmenting organisational involvement, as these practices might lower perceptions of qualitative job insecurity by clarifying one’s future role in the organisation [5]. In addition, interventions should be aimed at the mechanisms that underlie the relationship between qualitative job insecurity and learning, to buffer the negative consequences resulting from qualitative job insecurity.

First, a decline in occupational self-efficacy was responsible for lower levels of informal learning in terms of information-seeking and feedback-seeking from one’s supervisor and colleagues. Hence, organisations may want to increase employees’ occupational self-efficacy, for instance, by providing stress-management courses or online self-enhancement interventions, as prior research has indicated these are successful means to enhance self-efficacy [106,107].

Second, an increase in psychological contract breach was responsible for a decline in feedback-seeking from supervisors. Organisations might therefore benefit from implementing interventions that enhance employees’ fairness perceptions of their current work situation, such as enrolling employees in communication programs and encouraging employee participation in organisational change [57,108,109].

Finally, employers could invest in employees perceived employability, as this is viewed as a personal resource that provides an individual with a general feeling of control over his/her career [110]. Practitioners may try to enhance perceptions of employability by supporting career and skill development, such as the possibility to apply skills in a variety of contexts and the provision of training and development opportunities [111,112].

## 5. Conclusions

The results of this study highlight that qualitative job insecurity is an important stressor in the current workplace. Higher levels of qualitative job insecurity were related to lower levels of occupational self-efficacy, which resulted in a decrease in informal learning in terms of information-seeking, feedback-seeking from colleagues and feedback-seeking from one’s supervisor. In addition, qualitative job insecurity was related to perceived breach of the psychological contract, which translated itself in lower levels of feedback-seeking from one’s supervisor. Interventions might be aimed at increasing employees’ occupational self-efficacy and restoring the psychological contract, to prevent qualitatively job-insecure workers from withdrawing from informal learning.

## Figures and Tables

**Table 1:** Means, standard deviations, and correlations for the study variables.

**Variables**	**Means**	**SD**	**1**	**2**	**3**	**4**	**5**	**6**	**7**	**8**	**9**	**10**	**11**	**12**	**13**	**14**	**15**	**16**	**17**	**18**
1. Gender	0.81	0.37	-																	
2. Age	41.02	10.78	−0.07	-																
3. Low edu.	0.20	0.40	−0.05	0.12 **	-															
4. Uni.	0.13	0.33	−0.08 **	−0.12 **	−0.20 **	-														
5. QLJI T1	3.04	0.85	−0.03	−0.03	−0.01	−0.02	-													
6. QLJI T2	3.06	0.86	−0.05	−0.07	−0.09 *	−0.07	0.65 **	-												
7. QLJI T3	3.10	0.87	−0.02	−0.10 *	−0.09 *	−0.08	0.60 **	0.71 **	-											
8. OSE T1	3.80	0.51	−0.06 *	0.02	0.06 *	−0.02	−0.32 **	−0.25 **	−0.21 **	-										
9. OSE T2	3.81	0.49	−0.08	0.07	0.08	0.00	−0.30 **	−0.39 **	−0.31 **	0.52 **	-									
10. OSE T3	3.81	0.51	−0.04	−0.09 *	0.07	−0.01	−0.31 **	−0.31 **	−0.41 **	0.47 **	0.54 **	-								
11. PCB T1	2.64	0.80	0.02	0.02	−0.02	−0.03	0.35 **	0.29 **	0.19 **	−0.26 **	−0.32 **	−0.13 **	-							
12. PCB T2	2.88	0.77	0.08	−0.02	0.01	−0.07	0.27 **	0.37 **	0.24 **	−0.28 **	−0.37 **	−0.22 **	0.63 **	-						
13. PCB T3	2.60	0.74	0.06	−0.02	0.09 *	−0.07	0.26 **	0.34 **	0.30 **	−0.22 **	−0.29 **	−0.23 **	0.56 **	0.65 **	-					
14. ISB T1	4.18	0.56	0.09 *	−0.08 **	−0.13 **	0.11 **	−0.07 *	0.04	−0.03	0.19 **	0.17 **	0.20 **	−0.11 **	−0.10 *	−0.08 *	-				
15. ISB T2	4.18	0.52	0.02	−0.01	−0.07	0.11 **	−0.07	0.04	−0.02	0.16 **	0.22 **	0.20 **	−0.14 **	−0.14 **	−0.09	0.52 **	-			
16. ISB T3	4.21	0.50	0.07	−0.03	−0.04	0.07	−0.07	0.04	−0.03	0.22 **	0.19 **	0.24 **	−0.11 **	−0.17 **	−0.11 **	0.50 **	0.42 **	-		
17. FS coll. T1	3.77	0.73	0.10 **	−0.15 **	−0.03	0.03	−0.09 **	0.06	−0.07	0.16 **	0.16 **	0.10 *	−0.12 **	−0.10 *	−0.04	0.24 **	0.15 **	0.21 **	-	
18. FS coll. T2	3.74	0.73	0.10 *	−0.14 **	0.02	0.08	−0.09 *	0.18 **	−0.11 *	0.18 **	0.24 **	0.14 **	−0.11 **	−0.13 **	−0.10 *	0.18 **	0.21 **	0.22 **	0.57 **	-
19. FS coll. T3	3.78	0.69	0.13 **	−0.15 **	0.02	0.01	−0.08	0.02	−0.05	0.15 **	0.13 **	0.12 **	−0.05	−0.04	−0.05	0.14 **	0.18 **	0.28 **	0.53 **	0.49 **
20. FS sup. T1	3.48	0.84	0.04	0.05	0.04	−0.01	−0.21 **	−0.16 **	−0.15 **	0.17 **	0.17 **	0.11 **	−0.19 **	−0.15 **	−0.07	0.27 **	0.14 **	0.17 **	0.38 **	0.29 **
21. FS sup. T2	3.48	0.82	0.07	0.06	0.10 *	0.03	−0.29 **	−0.29 **	−0.25 **	0.18 **	0.31 **	0.21 **	−0.24 **	−0.20 **	−0.13 *	0.18 **	0.26 **	0.22 **	0.28 **	0.44 **
22. FS sup. T3	3.49	0.86	0.07	0.11 *	0.05	0.05	−0.16 **	−0.18 **	−0.22 **	0.16 **	0.23 **	0.18 **	−0.18 **	−0.21 **	−0.13 **	0.15 **	0.18 **	0.17 **	0.18 **	0.34 **
23. HSB T1	4.06	0.59	0.17 **	−0.14 **	−0.02	0.00	−0.09 **	−0.06	−0.03	0.10 **	0.13 **	0.09 *	−0.13 **	−0.05	−0.08	0.29 **	0.20 **	0.23 **	0.43 **	0.35 **
24. HSB T2	4.03	0.58	0.20 **	−0.07	0.04	−0.02	−0.04	−0.04	−0.05	0.14 **	0.11 **	0.12 *	−0.09 *	−0.08	−0.09	0.20 **	0.31 **	0.25 **	0.32 **	0.44 **
25. HSB T3	4.03	0.53	0.16 **	−0.09 *	−0.02	−0.02	−0.08	−0.02	−0.03	0.12 **	0.08	0.14 **	−0.12 **	−0.09	−0.09 *	0.16 **	0.19 **	0.33 **	0.33 **	0.25 **
**Variables**	**19**	**20**	**21**	**22**	**23**	**24**	**25**													
19. FS coll. T3	-																			
20. FS sup. T1	0.21 **	-																		
21. FS sup. T2	0.25 **	0.57 **	-																	
22. FS sup. T3	0.33 **	0.56 **	0.64 **	-																
23. HS T1	0.29 **	0.21 **	0.19 **	0.12 **	-															
24. HS T2	0.28 **	0.14 **	0.25 **	0.16 **	0.52 **	-														
25. HS T3	0.45 **	0.16 **	0.16 **	0.26 **	0.49 **	0.43 **	-													

Note: *N* = 1433; * *p* < 0.05, and ** *p* < 0.01; Low edu. = no degree of higher education; Uni. = university degree; QLJI = qualitative job insecurity; OSE = occupational self-efficacy; PCB = psychological contract breach; ISB = information-seeking behavior; FS coll. = feedback-seeking from colleagues; FS sup. = feedback-seeking from supervisors; HSB = help-seeking behavior.

**Table 2:** Fit indices of competing nested factor models, and standardized maximum likelihood estimates.

Model No.	Model	χ^2^	df	RMSEA	SRMR	CFI	∆CFI	TLI	Comparison to Model No.	Satorra–Bentler Corrected ∆χ^2^
Factorial structure of measurement model										
1	Six-factor model (hypothesized)	4087.474	1497	0.035	0.054	0.906		0.889		
2	One-factor model	15,412.05	1647	0.076	0.159	0.503	0.40	0.466	1	9032.74 **
3	Four-factor model (information-seeking, feedback-seeking, and help-seeking load on same factor)	6820.316	1584	0.048	0.091	0.811	0.10	0.789	1	2544.25 **
4	Seven-factor model (feedback seeking divided in feedback seeking from supervisor and from colleagues)	2313.664	1440	0.021	0.037	0.968	0.06	0.961	1	1773.81 **
Measurement invariance of seven-factor measurement model										
5	Metric invariance	2357.337	1466	0.021	0.040	0.968	0	0.961	4	43.34 *
6	Strong invariance	2441.884	1492	0.021	0.041	0.966	−0.002	0.959	5	88.90 **
7	Strict invariance	2511.546	1532	0.021	0.043	0.965	−0.001	0.959	6	67.29 **
8	Full invariance	2554.283	1552	0.021	0.043	0.964	−0.001	0.959	7	40.04 **

Note: *N* = 1433; all models fitted using a robust maximum likelihood estimator; * *p* < 0.05; ** *p* < 0.01.; RMSEA = root mean squared error of approximation; SRMR = standardized root mean squared residual; CFI = Comparative Fit Index; TLI = Tucker-Lewis Index.

**Table 3:** Test of alternative models and time invariance.

Model No.	Model	χ^2^	df	RMSEA	SRMR	CFI	∆CFI	TLI	Comparison to Model No.	Satorra–Bentler Corrected ∆χ^2^
Competing models of cross-lagged relationships										
1	Stability model	3635.24	1890	0.026	0.08	0.938		0.934		
2	Normal causation model	3529.514	1865	0.025	0.064	0.941	0.003	0.937	1	107.53 **
3	Reversed causation model	3588.833	1865	0.026	0.075	0.939	0.001	0.934	1	46.47 **
4	Reciprocal model	3504.213	1841	0.025	0.061	0.914	0.03	0.936	2	26.61
Time invariance of normal causation model										
5	Baseline model (all causal paths free to differ across time)	3529.514	1865	0.025	0.064	0.941		0.064		
6	Autoregressive paths fixed equal across time	3544.055	1872	0.025	0.064	0.941	0	0.064	5	14.17 *
7	Paths from QLJI → OSE and PCB fixed equal across time	3543.870	1874	0.025	0.064	0.941	0	0.064	6	0.12
8	Paths from OSE and PCB → informal learning fixed equal across time	3546.642	1882	0.025	0.064	0.941	0	0.064	7	3.79

Note: *N* = 1433; all models fitted using a robust maximum likelihood estimator; QLJI = qualitative job insecurity; OSE = occupational self-efficacy; PCB = psychological contract breach; * *p* < 0.05; ** *p* < 0.01.
